# Forecasting the COVID-19 Epidemic by Integrating Symptom Search Behavior Into Predictive Models: Infoveillance Study

**DOI:** 10.2196/28876

**Published:** 2021-08-11

**Authors:** Alessandro Rabiolo, Eugenio Alladio, Esteban Morales, Andrew Ian McNaught, Francesco Bandello, Abdelmonem A Afifi, Alessandro Marchese

**Affiliations:** 1 Department of Ophthalmology Gloucestershire Hospitals NHS Foundation Trust Cheltenham United Kingdom; 2 Department of Chemistry University of Turin Turin Italy; 3 Jules Stein Eye Institute David Geffen School of Medicine University of California Los Angeles Los Angeles, CA United States; 4 School of Health Professions Faculty of Health University of Plymouth Plymouth United Kingdom; 5 Department of Ophthalmology Vita-Salute University, IRCCS Ospedale San Raffaele Scientific Institute Milan Italy; 6 Department of Biostatistics Fielding School of Public Health University of California Los Angeles Los Angeles, CA United States

**Keywords:** Google Trends, symptoms, coronavirus, SARS-CoV-2, big data, time series, predictive models, Shiny web application, infodemiology, infoveillance, digital health, COVID-19

## Abstract

**Background:**

Previous studies have suggested associations between trends of web searches and COVID-19 traditional metrics. It remains unclear whether models incorporating trends of digital searches lead to better predictions.

**Objective:**

The aim of this study is to investigate the relationship between Google Trends searches of symptoms associated with COVID-19 and confirmed COVID-19 cases and deaths. We aim to develop predictive models to forecast the COVID-19 epidemic based on a combination of Google Trends searches of symptoms and conventional COVID-19 metrics.

**Methods:**

An open-access web application was developed to evaluate Google Trends and traditional COVID-19 metrics via an interactive framework based on principal component analysis (PCA) and time series modeling. The application facilitates the analysis of symptom search behavior associated with COVID-19 disease in 188 countries. In this study, we selected the data of nine countries as case studies to represent all continents. PCA was used to perform data dimensionality reduction, and three different time series models (error, trend, seasonality; autoregressive integrated moving average; and feed-forward neural network autoregression) were used to predict COVID-19 metrics in the upcoming 14 days. The models were compared in terms of prediction ability using the root mean square error (RMSE) of the first principal component (PC1). The predictive abilities of models generated with both Google Trends data and conventional COVID-19 metrics were compared with those fitted with conventional COVID-19 metrics only.

**Results:**

The degree of correlation and the best time lag varied as a function of the selected country and topic searched; in general, the optimal time lag was within 15 days. Overall, predictions of PC1 based on both search terms and COVID-19 traditional metrics performed better than those not including Google searches (median 1.56, IQR 0.90-2.49 versus median 1.87, IQR 1.09-2.95, respectively), but the improvement in prediction varied as a function of the selected country and time frame. The best model varied as a function of country, time range, and period of time selected. Models based on a 7-day moving average led to considerably smaller RMSE values as opposed to those calculated with raw data (median 0.90, IQR 0.50-1.53 versus median 2.27, IQR 1.62-3.74, respectively).

**Conclusions:**

The inclusion of digital online searches in statistical models may improve the nowcasting and forecasting of the COVID-19 epidemic and could be used as one of the surveillance systems of COVID-19 disease. We provide a free web application operating with nearly real-time data that anyone can use to make predictions of outbreaks, improve estimates of the dynamics of ongoing epidemics, and predict future or rebound waves.

## Introduction

COVID-19 is a new entity, and the dynamics of its propagation are difficult to predict. In the absence of compelling evidence, health and political decisions have been strongly driven by a wide variety of statistical models and simulation scenarios to forecast the COVID-19 epidemic. Still, large variations exist among the different models with respect to the predicted number of infected people, time to reach a peak of new cases, course of the epidemic, and identification of outbreaks [1]. One key limitation of such models is that they rely heavily on the number of confirmed infected subjects who usually seek medical attention due to moderate to severe symptoms. However, confirmed cases are most likely only a small proportion of the true number of cases as the vast majority of infected individuals are asymptomatic or mildly symptomatic [2].

There is increasing interest in the potential of “big data” analysis to predict future areas of COVID-19 outbreaks and incidence of cases based on symptom search behaviors. In the past, search query data have been used to facilitate early detection and near real-time estimates of flu and Dengue [3]. A few studies have shown a correlation between Google Trends data of medical term searches and COVID-19 metrics [4], suggesting that incorporating Google Trends data into conventional metrics could lead to better nowcasting and forecasting of the COVID-19 epidemic.

In this study, we systematically evaluate patterns of web queries for COVID-19 clinical manifestations and develop an open-access web application for exploring their correlations with COVID-19 propagation. We implement models integrating conventional COVID-19 metrics with Google Trends data and compare them to those not containing Google Trends data. The aim of this study is to present a framework for digital surveillance of COVID-19 using open-access big data of Google searches of symptoms associated with COVID-19.

## Methods

### Data Collection

Daily new confirmed COVID-19 cases, the cumulative number of COVID-19 cases, and the number of cases and deaths per million for all available countries were exported from the COVID-19 Data Repository by the Center for System Science and Engineering at Johns Hopkins University [5]. The selected countries used as case studies are given in the results section below. Country choice was arbitrary, and the following principles were adopted: representation of the five continents; inclusion of countries where the COVID-19 epidemic had different levels of severity and different evolutions over time; inclusion of countries where Google is the preferred search engine; exclusion of countries with limited access to the internet; exclusion of countries where one or more Google Trends topic had only zero or missing values in the selected time frame; and exclusion of countries whose reliability in terms of data reporting has been questioned. As data were fully anonymized and publicly available, no ethical approval was required.

The Google Trends application programming interface (API) was used to extract trends of Google searches for the most common COVID-19 signs and symptoms. For each search term, geographic region, and time frame selected, Google Trends outputs an “interest-over-time” index, which estimates the relative search volume on a normalized scale from 0 (no searches) to 100 (search term popularity peak). A total of 20 topics were identified on the basis of the most frequent signs and symptoms of COVID-19 and included the following: abdominal pain, ageusia, anorexia, anosmia, bone pain, chills, conjunctivitis, cough, diarrhea, eye pain, fatigue, fever, headache, myalgia, nasal congestion, nausea, rhinorrhea, shortness of breath, sore throat, and tearing [6-9]. Google Trends queries were carried out with the “topic” function, which includes all the related terms sharing the same concept in different languages. This approach ensures that the frequency of searches for closely related symptom types are appropriately grouped together.

For each country and search term, data were automatically exported as CSV files for two prespecified time frames: (1) five years of weekly data from July 1, 2015, to July 1, 2020, to study the long-term pattern of search terms, and (2) daily data from January 22, 2020, to December 20, 2020. As Google Trends allows daily data exportation up to 9 months, daily data were reconstructed by means of an overlapping method [10].

### Data Analysis

Interest-over-time values for the 5-year interval were used to distinguish topics with a significant deviation from their long-term pattern from the onset of the COVID-19 epidemic. For seasonal queries, trends were isolated from seasonal and random components with an additive decomposition method (Figure S1 in Multimedia Appendix 1); for nonseasonal queries, trends were extracted by smoothing the time series with a 1-year moving average. Decomposition plots were visually inspected, and topics with no clear change in their 5-year trends from January 2020 were excluded from the subsequent analyses.

The relationship between the daily interest-over-time values for the selected topics and COVID-19 confirmed deaths and new cases were investigated in the shorter time frame indicated above. Relationships between interest-over-time values of each topic and the number of new daily confirmed cases or deaths per million were visually assessed with line graphs. Changes in interest-over-time values over time were visually assessed with streamgraphs. To smooth daily fluctuations in both interest-over-time values and number of new cases, plots were generated using a 7-day moving average.

Time-lagged cross-correlations between COVID-19 new cases and each topic were calculated, using a 7-day moving average of both interest-over-time values and COVID-19 confirmed cases and deaths to blunt the day-by-day fluctuation.

### Model Development and Assessment

Principal component analysis (PCA) was used to perform data dimensionality reduction, decrease the number of input variables, and filter out noisy or redundant information. For each country, two PCA models were implemented: one using unprocessed data and the other using 7-day moving average smoothing. PCA was applied to standardized data (ie, with zero mean and unit variance). The PCA model was graphically inspected through PCA score and loading plots. PCA was assessed via 5-fold cross-validation, and the results obtained in each test sample were averaged. The amount of variance explained by each principal component (PC) in the model was inspected with scree plots. Based on the elbow and Kaiser rules, the first two PCs (PC1 and PC2) were subsequently used for time series modelling [11].

A total of three different time series models were fitted on PC1 and PC2 values: error, trend, seasonality (ETS); autoregressive integrated moving average (ARIMA); and a feed-forward neural network autoregression (NNAR) model with one hidden layer [12]. Models were fitted on a 30-day window and used to predict future PC1 and PC2 values up to 14 days. The 14th predicted day was aligned to the peak and base of each wave. The new data scores predicted with the time series models were then reinserted into the model as input variables.

For each country, the three models were compared in terms of ability to predict the PC1 and PC2 using the root mean square error (RMSE) of the predicted values. For each time series model, the predictive abilities of the model generated with raw data and the one generated with 7-day moving averages were compared. To further assess the PCA models based on both Google Trends data and conventional COVID-19 metrics, we also generated predictive models based on conventional COVID-19 metrics only; we then compared the predictive ability of models with and without Google Trends data by means of RMSE for each country.

### Web Application

An open-source web application was developed using R Shiny [13]. Data are collected, imported, and updated daily for 188 countries from the sources mentioned above.

The web application allows users to generate line graphs and streamgraphs to visualize interest-over-time values and COVID-19 metrics and view worldwide trends over time in a choropleth map. Relationships between the variables at various lags can be explored with cross-correlations. The web application allows fitting and evaluating PCA models, fitting a time series model (either ETS or ARIMA), predicting PC components or any of the input variables of the model (including numbers of new cases and deaths), and evaluating the model performance graphically and with various metrics, such as RMSE and mean absolute error. The user has operational control of several model features, including the subset of variables to build the PCA model, the time window to fit the time series model, and the time interval to predict.

### Data Sharing

Raw data used in this study are publicly available on the COVID-19 Data Repository by the Center for System Science and Engineering at Johns Hopkins University [14] and the Google Trends webpage [15]. All processed data used in this study can be accessed and reproduced on the PredictPandemic website [13]. The code for the current version of the web application is open source and freely available [16].

## Results

**Figure 1:**
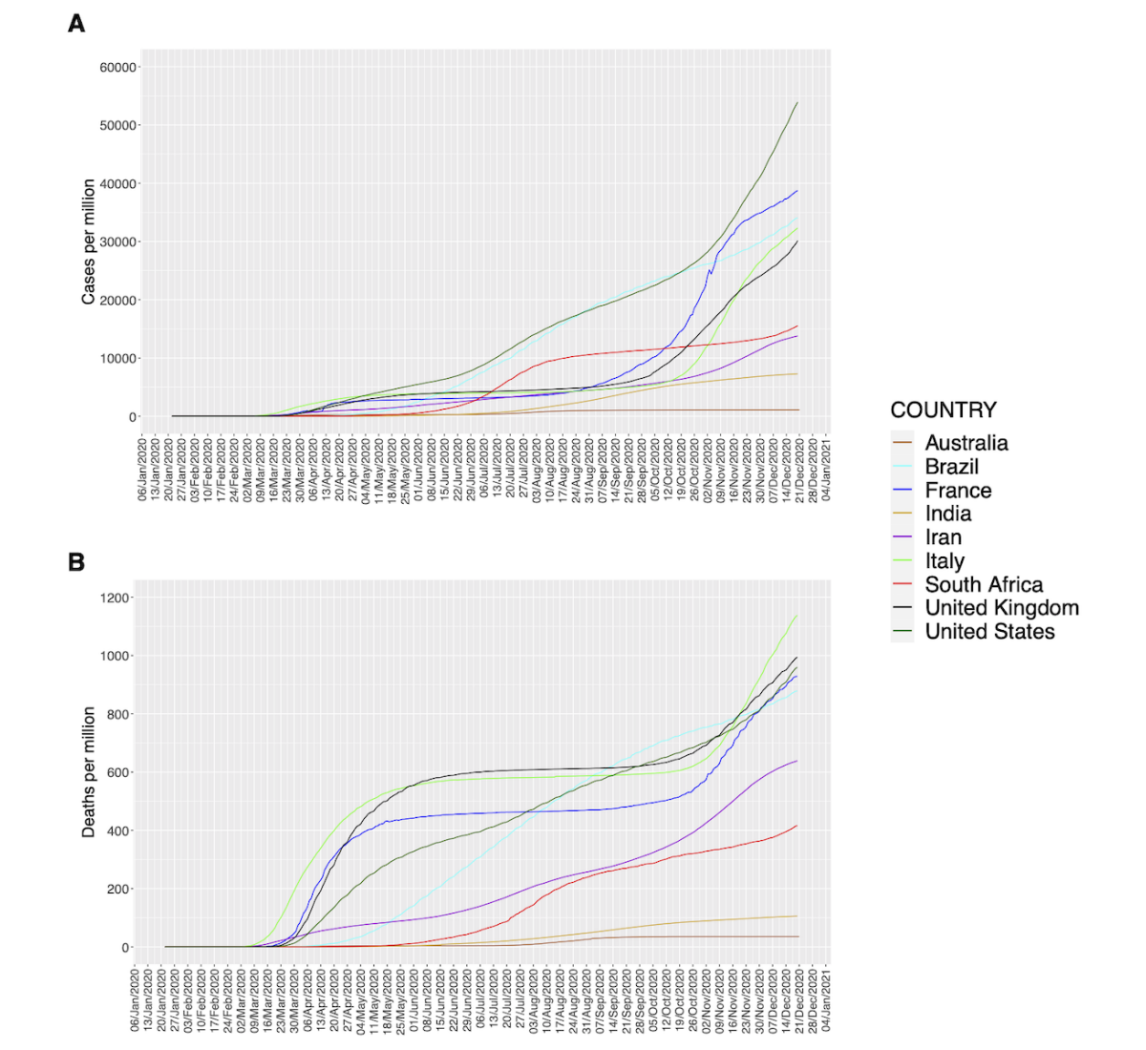
Cumulative number of confirmed cases (A) and deaths (B) per million for each country over time.

For this study, three European countries (Italy, United Kingdom, and France), one Asian country (India), one Oceanian country (Australia), one North American country (United States), one South American country (Brazil), one African country (South Africa), and one Middle Eastern country (Iran) were chosen as case studies. The cumulative numbers of cases and deaths in the selected countries are illustrated in Figure 1.

Table 1 summarizes information from the 5-year analysis. Among the 20 screened topics, 13 showed seasonality, while the remaining were nonseasonal. Overall, 11 search topics (Figures S2-S20 in Multimedia Appendix 1) showed a clear deviation from their 5-year trend: ageusia, anosmia, chills, cough, eye pain, fever, headache, nasal congestion, rhinorrhea, shortness of breath, and sore throat.

**Table 1:** Symptoms screened in the 5-year analysis.

Topic	Seasonality	Deviation from 5-year trend^a^
Abdominal pain	Nonseasonal	No
Ageusia	Nonseasonal	Yes
Anorexia	Seasonal	No
Anosmia	Nonseasonal	Yes
Bone pain	Nonseasonal	No
Chills	Seasonal	Yes
Conjunctivitis	Seasonal	No
Cough	Seasonal	Yes
Diarrhea	Seasonal	No
Eye pain	Nonseasonal	Yes
Fatigue	Seasonal	No
Fever	Seasonal	Yes
Headache	Seasonal	Yes
Myalgia	Seasonal	No
Nasal congestion	Seasonal	Yes
Nausea	Nonseasonal	No
Rhinorrhea	Seasonal	Yes
Shortness of breath	Seasonal	Yes
Sore throat	Seasonal	Yes
Tearing	Nonseasonal	No

^a^Topic categorization into deviating and not deviating from their 5-year trend was determined on the visual inspection of decomposition plots.

The relationships between the number of new cases and each search topic are illustrated in Figures S21-S29 in Multimedia Appendix 1. Several symptoms, including ageusia, anosmia, cough, rhinorrhea, and sore throat were aligned with the COVID-19 epidemic in most countries and were searched on Google well before the number of COVID-19 confirmed cases peaked. On the other hand, other topics showed less evident variations (chills, eye pain) or deviated from their trend only during the first wave (headache, shortness of breath). Additionally, the peak of interest in all symptoms (except eye pain) preceded that of confirmed COVID-19 cases in most countries, and topics increasing earlier reached their highest interest-over-time value before those growing later. Similar patterns were observed for interest-over-time of search terms when compared to the number of newly confirmed deaths (Figures S30-S38 in Multimedia Appendix 1).

**Figure 2:**
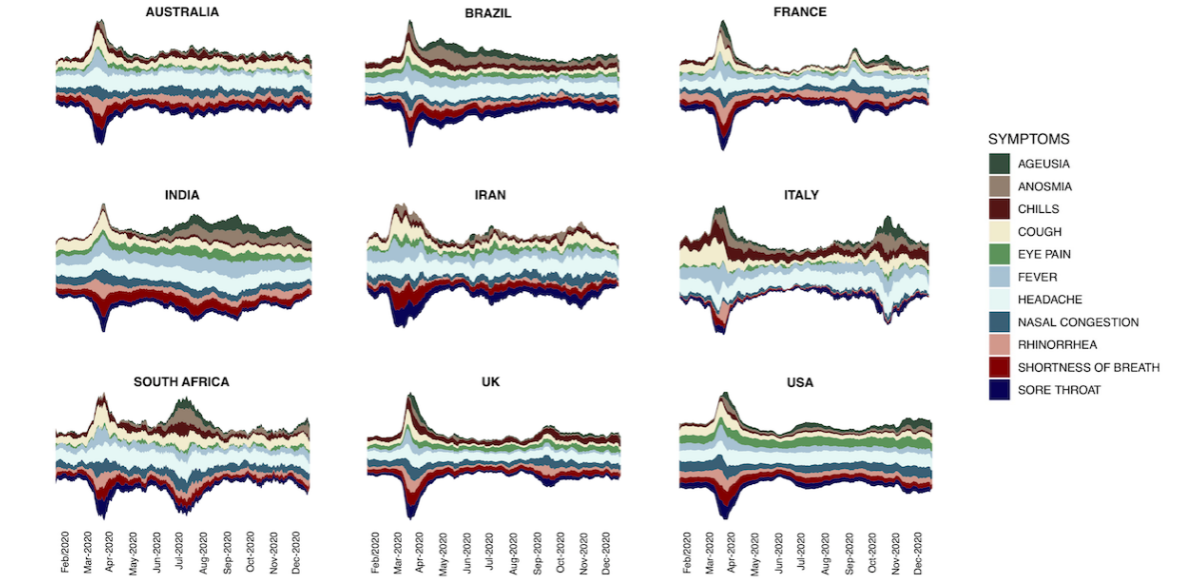
Streamgraphs of the interest-over-time index for each individual country. The x-axis values are given in months. Index-over-time values were plotted as 7-day moving average.

The interest-over-time change for all topics is illustrated in Figure 2. Overall, the interest-over-time values of the selected topics had a peak in March 2020 in all selected countries. In Italy, France, and South Africa—and, to a lesser extent, Iran, the United Kingdom, and the United States—there was a decrease in the search terms after the first peak, followed by a second peak. In Iran, a third peak in searches was seen, corresponding to the third COVID-19 wave. In India and Brazil, searches of medical terms remained high after the first peak, and no second peak was seen. In Australia, the interest-over-time values of the selected topics returned to the pre-peak values soon after the first peak in March and remained low and stable.

Cross-correlations between each topic and the number of confirmed COVID-19 cases are reported in Tables S1-S9 in Multimedia Appendix 1. Overall, ageusia, anosmia, and headache were most consistently correlated with COVID-19 cases across the selected countries. The degree of correlation and the best time lag largely varied as a function of the selected country and topic, but overall the optimal time lag was 15 days.

The scores and loadings plots for the PCA models are given in Figures S39-S47 in Multimedia Appendix 1. The scores plot represents a summary of the collected data trends over time, while the loadings plot shows how strongly each variable influences a PC. In the month of March 2020, all selected countries deviated considerably from their previous scores and moved toward the PCs directions of the loadings of the Google search terms, preceding the increment in the number of searches of the symptoms related to COVID-19. The latest 14 days show a similar pattern for all selected countries, pointing toward the loadings' directions of deaths and new cases. Specifically, for Italy, France, and South Africa, the latest scores are in the same area of loadings plots corresponding to deaths and new cases, indicating a stable trend in these metrics. On the other hand, the United Kingdom, the United States, Brazil, and India followed a worsening pattern, as their scores kept moving toward the direction of new deaths and cases. Australia and Iran were the only selected countries showing an improving trend, with the score points moving away from the loadings of COVID-19 deaths and new cases.

**Figure 3:**
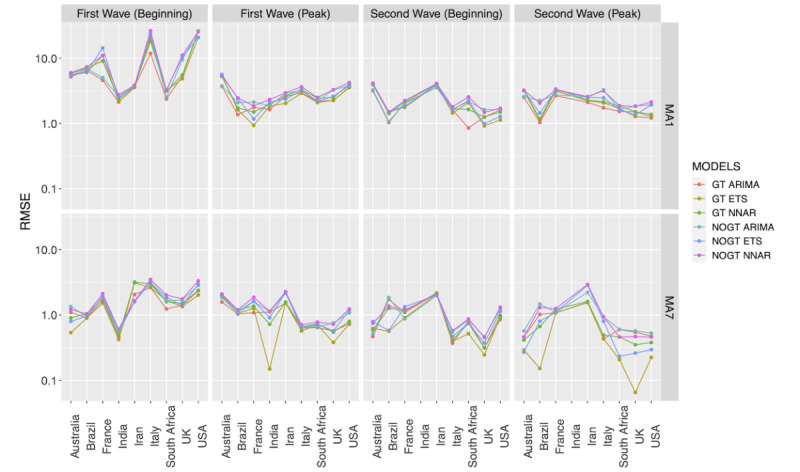
Root mean square errors of the prediction error for principal component 1 of the various models for the selected countries. MA1 and MA7 indicate analyses performed on 1-day (ie, original data) and 7-day moving averages of data, respectively. GT indicates models based on both traditional COVID-19 metrics and Google Trends data, while NOGT models are based on COVID-19 metrics only. ARIMA: autoregressive integrated moving average; ETS: error, trend, seasonality; NNAR: feed-forward neural network autoregression; RMSE: root mean square error; UK: United Kingdom; USA: United States of America.

Figure 3 illustrates the RMSE for the prediction of PC1 values with the three time series models in the various countries using raw data and a 7-day moving average. Models based on the 7-day moving average lead to considerably smaller RMSE values as opposed to those calculated with raw data (median 0.90, IQR 0.50-1.53 versus median 2.27, IQR 1.62-3.74, respectively). Overall, predictions based on both search terms and COVID-19 conventional metrics performed better than those not including Google searches (median 1.56, IQR 0.90-2.49 versus median 1.87, IQR 1.09-2.95, respectively), but the improvement in prediction varied as a function of the selected country and time frame. Although ETS (median 1.62, IQR 0.87-2.7) led to slightly smaller RMSE than ARIMA (median 1.65, IQR 1.04-2.58) and NNAR (median 1.82, IQR 1.15-3.15) models, none of the tested time series models clearly outperformed the remaining two, and the best model varied as a function of country, time range, and period of time selected. Similar results were obtained when trying to predict PC2 (Figure S48 in Multimedia Appendix 1).

## Discussion

In this study, we investigated the relationship between Google Trends searches of symptoms associated with COVID-19 and confirmed COVID-19 cases and deaths. We found that some of the search terms showed an unusually high recent online interest that deviated considerably from their expected behavior and preceded the peak of confirmed COVID-19 cases by days to weeks. This pattern was consistent across different countries and of similar magnitude. We developed and validated predictive models to forecast the COVID-19 epidemic based on the combination of Google Trends searches of symptoms associated with COVID-19 and traditional COVID-19 metrics. We found that models incorporating Google Trends data generally performed better than those based solely on traditional COVID-19 metrics. We also developed a web application [13] to translate our approach into action.

Our study identified patterns of Google searches of several symptoms and signs associated with COVID-19 in a consistent way across the studied countries. Overall, Google searches of COVID-19 symptoms followed a similar trend to that of the COVID-19 epidemic and preceded traditional COVID-19 metrics. This behavior can contribute to the early recognition of new waves and epidemic peaks.

The interpretation of symptom search behavior during COVID-19 outbreaks should be carefully considered. Dynamics of online searches may show atypical patterns during pandemics where major restrictions occur, including shutdowns of economic activities, movement restrictions, and health care overload [17]. Constant media attention may contribute to increasing interest in some of the studied topics [18]. COVID-19 received extensive coverage that might have precipitated unusually high interest during lockdowns [19]. Our findings on online search behavior might be secondary to general media interest in specific COVID-19 symptoms, rather than a primary, and possibly predictive, consequence of people with COVID-19 researching their own symptoms in real time. All the selected countries had a peak in searches of medical terms in or around March and April 2020, including those countries with low numbers of cases at that time, such as South Africa and India. This pattern may indicate that curiosity and media coverage regarding the new pandemic can explain part of the first peak in search terms, in agreement with a previous study [20]. After the first peak, however, Google search behavior followed different patterns across countries and resembled the course of the COVID-19 epidemic. In those countries that had a second wave—such as Italy, France, Iran, the United Kingdom, or the United States—the number of Google searches had a second peak; the height of the second peak of the searches was lower than that of the first peak, despite a higher number of reported cases and deaths, suggesting that individual curiosity regarding the new pandemic could have inflated the first peak in search terms. Iran also had a third peak in searches between October and December 2020, when the country had a third COVID-19 wave. South Africa had a second peak in its searches in July 2020, when the country had its first wave. In India, the first peak in searches was followed by a steady increase from June 2020, after which searches remained stable until October 2020, and then decreased gradually, resembling the shape of the COVID-19 epidemic in this country. In Australia, which effectively managed the COVID-19 epidemic and had among the lowest infection and death rates in the world, the interest-over-time of the various search terms after the first peak remained low and comparable to pre-peak values.

We observe that not all the selected topics reached their peak searches simultaneously; rather, they had different time patterns, which were fairly consistent across all countries. We believe that the intense and simultaneous media coverage of all the selected topics should have had the same effect at the same date if the media influence entirely caused this search behavior [21]. Ageusia and anosmia showed the highest correlations when lagged by a few days, while cough, fever, nasal congestion, sore throat, rhinorrhea, and shortness of breath preceded increases in COVID-19 cases by up to two weeks. This finding is consistent with the clinical course of COVID-19; in a large multicenter European study, olfactory and gustatory dysfunctions were among the latest and first manifestations in approximately 65% and 12% of patients, respectively [8].

In addition to describing how Google search terms changed over time in different countries and investigating their relationship with the numbers of cases and deaths, we also developed models combining interest-over-time values of searches of COVID-19 symptoms with conventional metrics (eg, number of new cases, number of new deaths) to predict the course of the COVID-19 epidemic, and we compared the prediction ability of these models against that of models based only on conventional metrics. The PCA approach allowed us to reduce dimensionality, summarize information into 2 PCs, and filter out the noisy or redundant information. Another advantage of PCA was to provide visual representations of data patterns, similarity trends, and outliers. The PCA approach is highly flexible and potentially allows accommodating new variables of interest in future versions of our application. As the PCA itself does not make any predictions, we processed the PC computed values with different time series models, and new data scores predicted with the time series models were reinserted into the PCA model. Our approach allows the extraction of the predicted values of any input variable, including the number of new cases and deaths. Models integrating interest-over-time values of the search topics and COVID-19 traditional metrics generally outperformed models based solely on confirmed cases and deaths, leading to improved predictions. There was no single best model in this study, and the best performing time series model varied as a function of the country, time frame, and moving average. Predictions were more accurate, leading to considerably smaller RMSE, when obtained using a 7-day moving average rather than daily data. This result is not surprising as Google Trends data have high daily fluctuations, and COVID-19 reported cases greatly oscillate, reflecting testing and reporting practices and contingencies [22,23].

To translate our results into practice so that the scientific community, agencies, and even curious users could potentially use them, we developed a freely available web application [13]. The application is interactive and updates the data daily, so it operates in near real time. It allows the user to visualize data for 188 countries, choosing any time frame. In addition, COVID-19 traditional metrics and Google search terms’ interest-over-time can be visualized globally on different graphs. The user can explore cross-correlations among selected keywords, generate predictive models with default variables or a user-selected subset of variables, and check model performance.

This study has limitations. The Google Trends algorithm is a “black box,” and the exact calculation formula for interest over time and raw data has never been made public. Search results may differ slightly when downloaded by different computers or on different days. However, we conducted search-research reliability, which showed excellent reliability for most of the topics included in this study (data not shown). The exclusion of those symptoms with no significant deviation from their 5-year trend reduced the possibility of spurious correlations, but it was not possible to account for seasonality in the selected topics. In other words, a small proportion of the increasing trend in some topics might be explained by their usual seasonal variations. The results of this study may not apply to countries where Google is not a popular search engine or where Google is censored or limited in its use. However, this approach can be applied to other search engines (eg, Baidu, Yahoo, Naver), as was done in previous studies on different diseases and on COVID-19 in Hubei province, China [24,25]. Previous studies have shown that self-reported symptoms on social media networks, such as Twitter, can provide useful information to track the COVID-19 pandemic and can be used for infoveillance along with search engine data [26-28]. Our approach could, in principle, be applied to social media as well. Geographical areas and groups of people (older adults and children) with scarce internet access cannot be studied with this strategy, and our results may not apply to largely rural countries. This study included only the most common clinical manifestations of COVID-19, and only a few selected countries were included as a case study. However, information and models for every country can be found on our web application.

Future work will include increased data granularity, allowing users to access information and make predictions at a regional level. Other metrics of interest, such as hospitalizations, will be included in our analysis as outcomes. Finally, we plan to allow the user to generate a one-page report for each country, summarizing the most relevant information.

In conclusion, the results of this study show that Google Trends searches during the COVID-19 pandemic may precede outbreaks by up to two weeks. The inclusion of digital online searches in statistical models may improve the nowcasting and forecasting of the COVID-19 epidemic and could be used as one of the surveillance systems employed by government agencies and supranational organizations to refine their monitoring of COVID-19. We provide a free web application operating with nearly real-time data that anyone can use to make predictions of outbreaks, improve estimates of dynamics of ongoing epidemics, and predict future or rebound waves.

## Appendix

Multimedia Appendix 1 Supplementary figures and tables.

## Abbreviations

ARIMA: autoregressive integrated moving average
ETS: error, trend, seasonality
NNAR: neural network autoregression
PC: principal component
PCA: principal component analysis
