# Supplementary material for: Forecasting the COVID-19 Epidemic by Integrating Symptom Search Behavior Into Predictive Models: Infoveillance Study
Source: J Med Internet Res. 2021 Aug 11;23(8):e28876. doi: 10.2196/28876 (PMC8360333; doi:10.2196/28876)
Supplement: Multimedia Appendix 1 [file jmir_v23i8e28876_app1.docx]

**Supplementary Appendix**

Supplementary information for term searches on Google Trends

Terms on Google Trends were searched using the «topic» function. In specific cases, Google Trends indicates the category of the topic, which is provided below between brackets for each term search included in the analysis:

- abdominal pain (syndrome)
- ageusia (topic)
- anorexia (symptom)
- anosmia (topic)
- bone pain (disease)
- chills (topic)
- conjunctivitis (topic)
- cough (topic)
- diarrhea (topic)
- eye pain (topic)
- fatigue (medical condition)
- fever (medical condition)
- headache (medical condition)
- myalgia (topic)
- nasal congestion (syndrome)
- nausea (disorder)
- rhinorrhea (medical condition)
- shortness of breath (disease)
- sore throat (topic)
- tearing (topic)

Figure S1. Example of the 5-year decomposition plot for the interest over time (IOT) of the seasonal topic cough in France. The observed time series (first row) was decomposed in its trend (second row), seasonal component (third row), and random fluctuations (fourth row).

Figure S2. 5-year extracted trend of the interest over time (IOT) of the topic abdominal pain in the various countries.

Figure S3. 5-year extracted trend of the interest over time (IOT) of the topic ageusia in the various countries.

Figure S4. 5-year extracted trend of the interest over time (IOT) of the topic anorexia in the various countries.

Figure S5. 5-year extracted trend of the interest over time (IOT) of the topic anosmia in the various countries.

Figure S6. 5-year extracted trend of the interest over time (IOT) of the topic bone pain in the various countries.

Figure S7. 5-year extracted trend of the interest over time (IOT) of the topic chills in the various countries.

Figure S8. 5-year extracted trend of the interest over time (IOT) of the topic conjunctivitis in the various countries.

Figure S9. 5-year extracted trend of the interest over time (IOT) of the topic cough in the various countries.

Figure S10. 5-year extracted trend of the interest over time (IOT) of the topic diarrhea in the various countries.

Figure S11. 5-year extracted trend of the interest over time (IOT) of the topic eye pain in the various countries.

Figure S12. 5-year extracted trend of the interest over time (IOT) of the topic fatigue in the various countries.

Figure S13. 5-year extracted trend of the interest over time (IOT) of the topic fever in the various countries.

Figure S14. 5-year extracted trend of the interest over time (IOT) of the topic headache in the various countries.

Figure S15. 5-year extracted trend of the interest over time (IOT) of the topic myalgia in the various countries.

Figure S16. 5-year extracted trend of the interest over time (IOT) of the topic nasal congestion in the various countries.

Figure S17. 5-year extracted trend of the interest over time (IOT) of the topic rhinorrhea in the various countries.

Figure S18. 5-year extracted trend of the interest over time (IOT) of the topic shortness of breath in the various countries.

Figure S19. 5-year extracted trend of the interest over time (IOT) of the topic sore throat in the various countries.

Figure S20. 5-year extracted trend of the interest over time (IOT) of the tearing in the various countries.

Figure S21. Line graphs showing the interest over time (IOT) of the selected topic searches (blue lines) and their relationship with the normalized number of incident cases per million people (histogram) in Australia. Data are plotted as a 7-day moving average to smooth day-by-day fluctuations.

Figure S22. Line graphs showing the interest over time (IOT) of the selected topic searches (blue lines) and their relationship with the normalized number of incident cases per million people (histogram) in Brazil. Data are plotted as a 7-day moving average to smooth day-by-day fluctuations.

Figure S23. Line graphs showing the interest over time (IOT) of the selected topic searches (blue lines) and their relationship with the normalized number of incident cases per million people (histogram) in France. Data are plotted as a 7-day moving average to smooth day-by-day fluctuations.

Figure S24. Line graphs showing the interest over time (IOT) of the selected topic searches (blue lines) and their relationship with the normalized number of incident cases per million people (histogram) in India. Data are plotted as a 7-day moving average to smooth day-by-day fluctuations.


Figure S25. Line graphs showing the interest over time (IOT) of the selected topic searches (blue lines) and their relationship with the normalized number of incident cases per million people (histogram) in Iran. Data are plotted as a 7-day moving average to smooth day-by-day fluctuations.

Figure S26. Line graphs showing the interest over time (IOT) of the selected topic searches (blue lines) and their relationship with the normalized number of incident cases per million people (histogram) in Italy. Data are plotted as a 7-day moving average to smooth day-by-day fluctuations.

Figure S27. Line graphs showing the interest over time (IOT) of the selected topic searches (blue lines) and their relationship with the normalized number of incident cases per million people (histogram) in South Africa. Data are plotted as a 7-day moving average to smooth day-by-day fluctuations.

Figure S28. Line graphs showing the interest over time (IOT) of the selected topic searches (blue lines) and their relationship with the normalized number of incident cases per million people (histogram) in the United Kingdom. Data are plotted as a 7-day moving average to smooth day-by-day fluctuations.

Figure S29. Line graphs showing the interest over time (IOT) of the selected topic searches (blue lines) and their relationship with the normalized number of incident cases per million people (histogram) in the United States. Data are plotted as a 7-day moving average to smooth day-by-day fluctuations.

Figure S30. Line graphs showing the interest over time (IOT) of the selected topic searches (blue lines) and their relationship with the normalized number of incident deaths per million people (histogram) in Australia. Data are plotted as a 7-day moving average to smooth day-by-day fluctuations.

Figure S31. Line graphs showing the interest over time (IOT) of the selected topic searches (blue lines) and their relationship with the normalized number of incident deaths per million people (histogram) in Brazil. Data are plotted as a 7-day moving average to smooth day-by-day fluctuations.

Figure S32. Line graphs showing the interest over time (IOT) of the selected topic searches (blue lines) and their relationship with the normalized number of incident deaths per million people (histogram) in France. Data are plotted as a 7-day moving average to smooth day-by-day fluctuations.

Figure S33. Line graphs showing the interest over time (IOT) of the selected topic searches (blue lines) and their relationship with the normalized number of incident deaths per million people (histogram) in India. Data are plotted as a 7-day moving average to smooth day-by-day fluctuations.

Figure S34. Line graphs showing the interest over time (IOT) of the selected topic searches (blue lines) and their relationship with the normalized number of incident deaths per million people (histogram) in Iran. Data are plotted as a 7-day moving average to smooth day-by-day fluctuations.

Figure S35. Line graphs showing the interest over time (IOT) of the selected topic searches (blue lines) and their relationship with the normalized number of incident deaths per million people (histogram) in Italy. Data are plotted as a 7-day moving average to smooth day-by-day fluctuations.

Figure S36. Line graphs showing the interest over time (IOT) of the selected topic searches (blue lines) and their relationship with the normalized number of incident deaths per million people (histogram) in South Africa. Data are plotted as a 7-day moving average to smooth day-by-day fluctuations.

Figure S37. Line graphs showing the interest over time (IOT) of the selected topic searches (blue lines) and their relationship with the normalized number of incident deaths per million people (histogram) in the United Kingdom. Data are plotted as a 7-day moving average to smooth day-by-day fluctuations.

Figure S38. Line graphs showing the interest over time (IOT) of the selected topic searches (blue lines) and their relationship with the normalized number of incident deaths per million people (histogram) in the United States. Data are plotted as a 7-day moving average to smooth day-by-day fluctuations.

Figure S39. Principal component analysis scores and loadings plot for Australia. The data starts from 22/Jan/2020 (red dot) to 20/Dec/2020 (Green cross); black dots over red lines indicate weeks.

Figure S40. Principal component analysis scores and loadings plot for Brazil. The data starts from 22/Jan/2020 (red dot) to 20/Dec/2020 (Green cross); black dots over red lines indicate weeks.


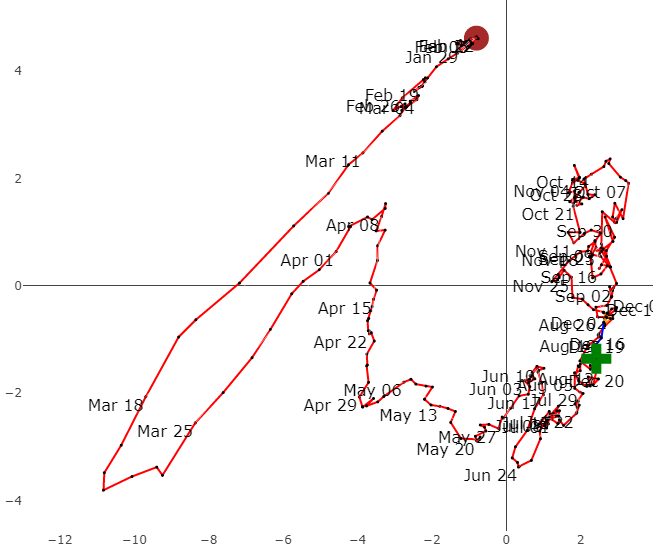

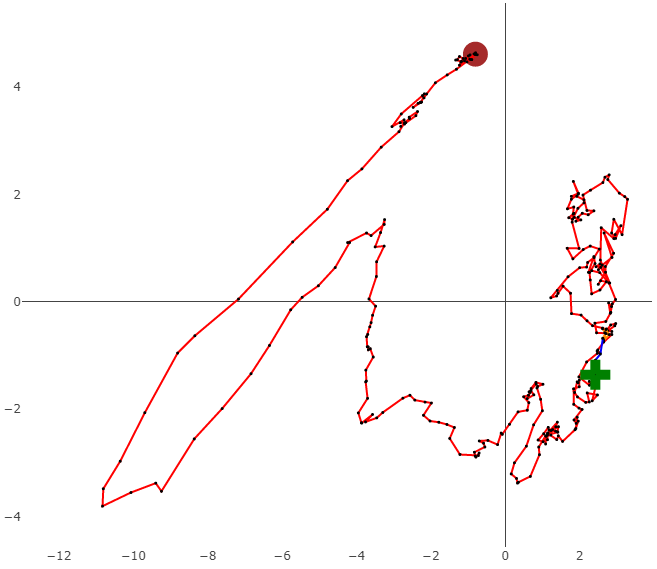

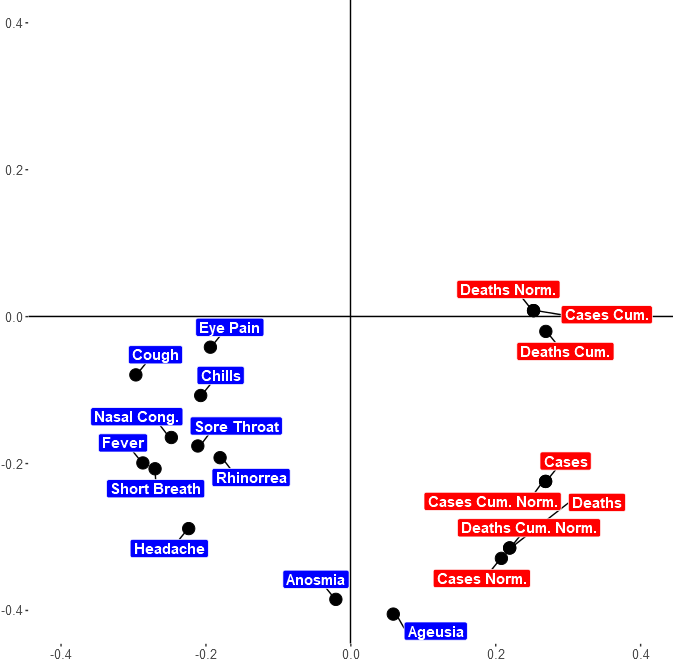


**Scores Plot**

**Component 1**

**Component 2**

**Loadings Plot**

**Component 1**

**Component 2**


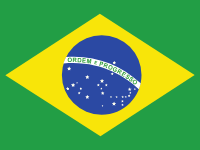


Figure S41. Principal component analysis scores and loadings plot for France. The data starts from 22/Jan/2020 (red dot) to 20/Dec/2020 (Green cross); black dots over red lines indicate weeks.


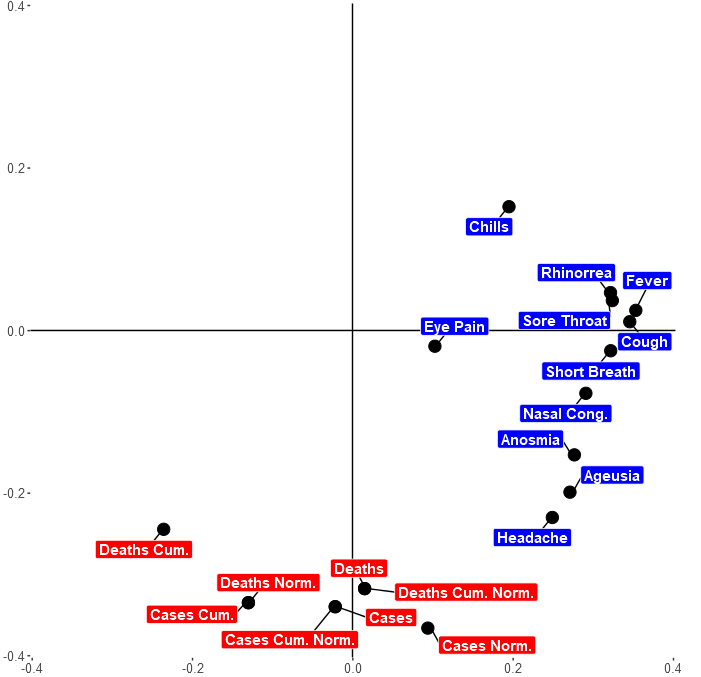


**Scores Plot**

**Component 1**

**Component 2**


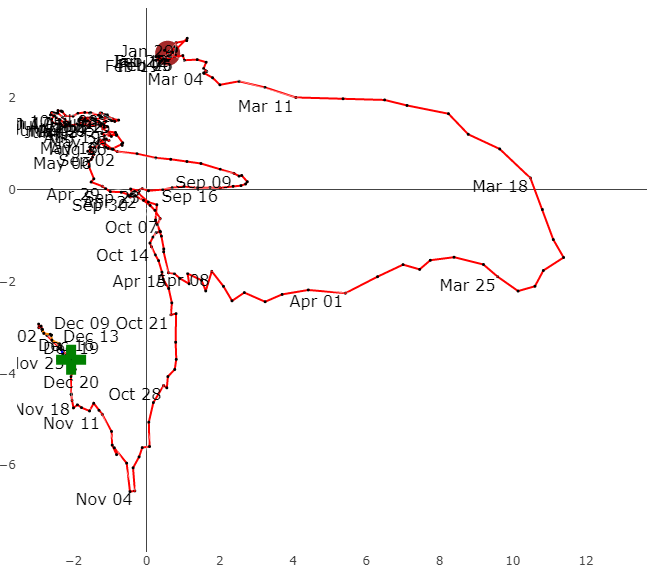


**Loadings Plot**

**Component 1**


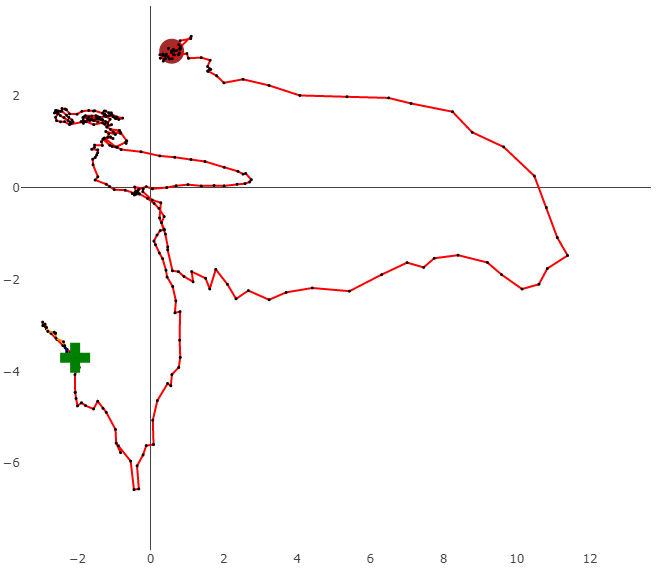


**Component 2**


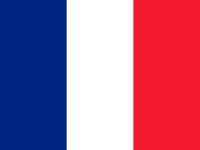


Figure S42. Principal component analysis scores and loadings plot for India. The data starts from 22/Jan/2020 (red dot) to 20/Dec/2020 (Green cross); black dots over red lines indicate weeks.

Figure S43. Principal component analysis scores and loadings plot for Iran. The data starts from 22/Jan/2020 (red dot) to 20/Dec/2020 (Green cross); black dots over red lines indicate weeks.


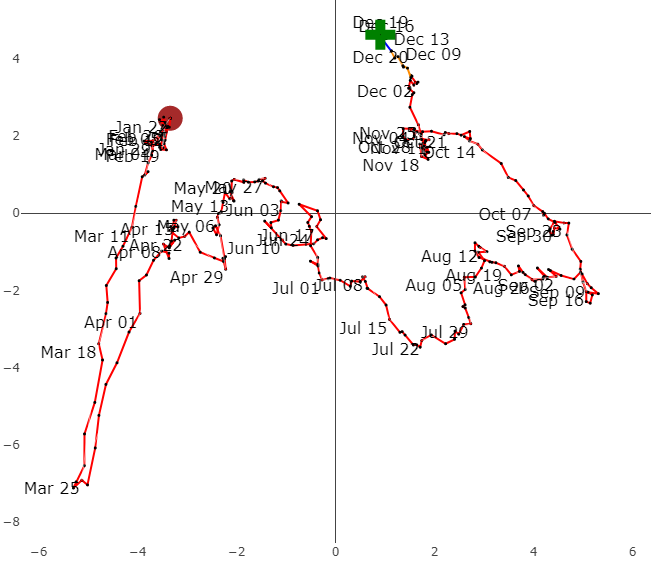

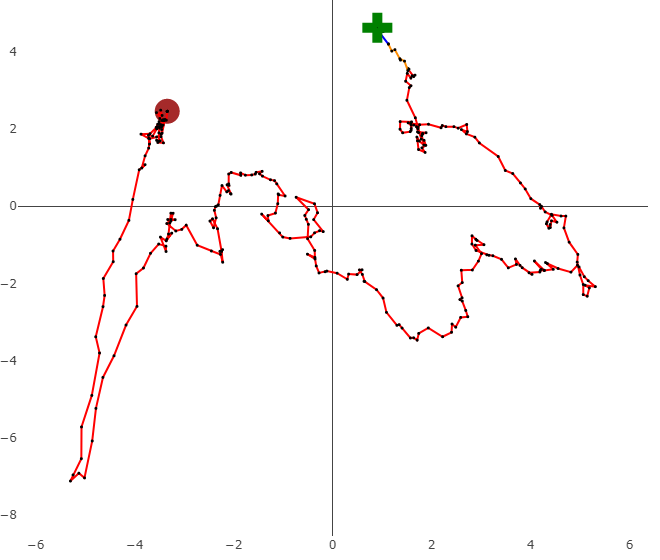

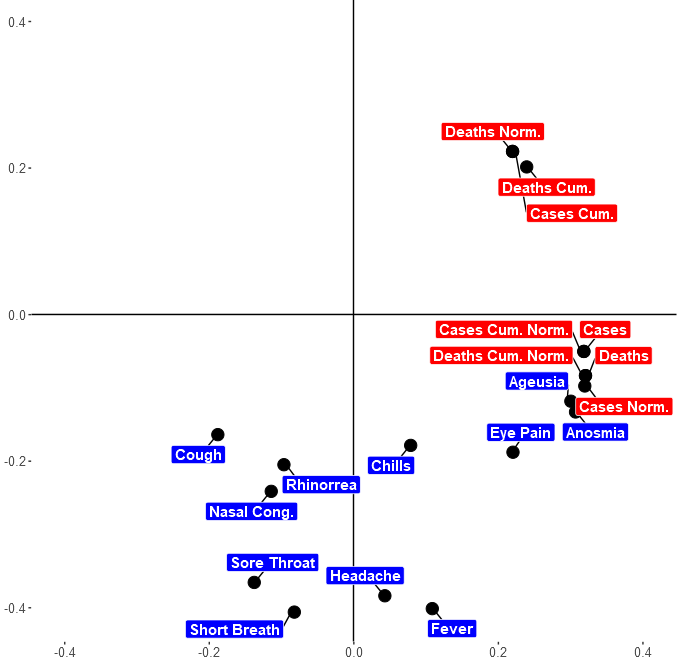


**Scores Plot**

**Component 1**

**Component 2**

**Loadings Plot**

**Component 1**

**Component 2**


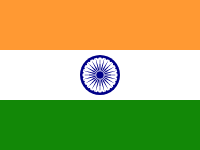

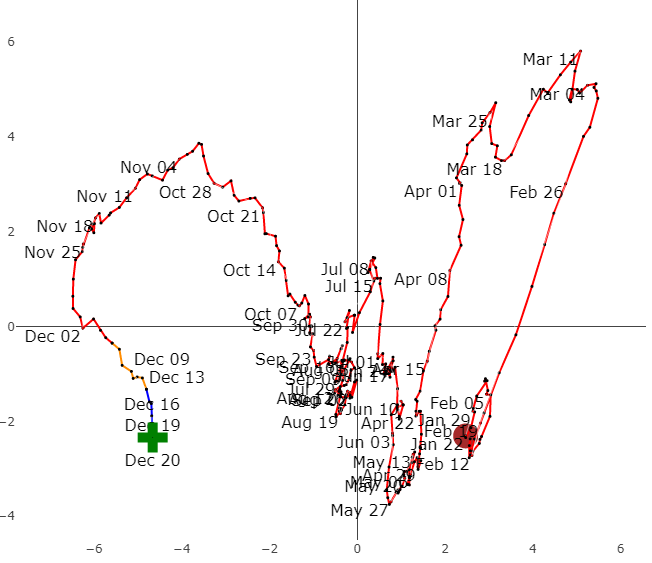

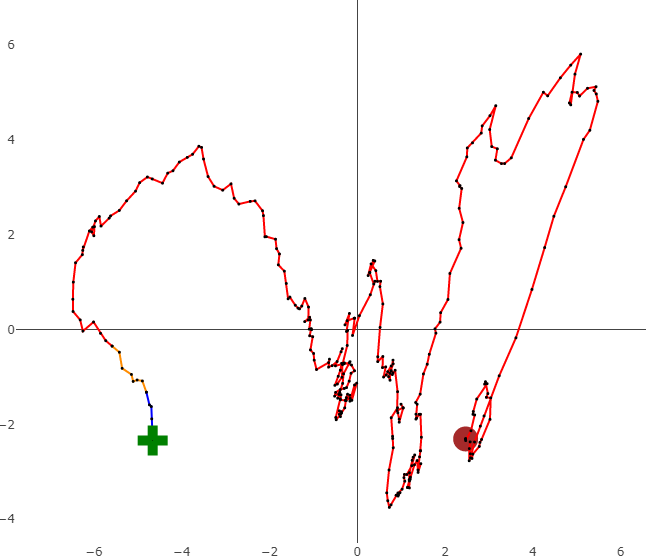

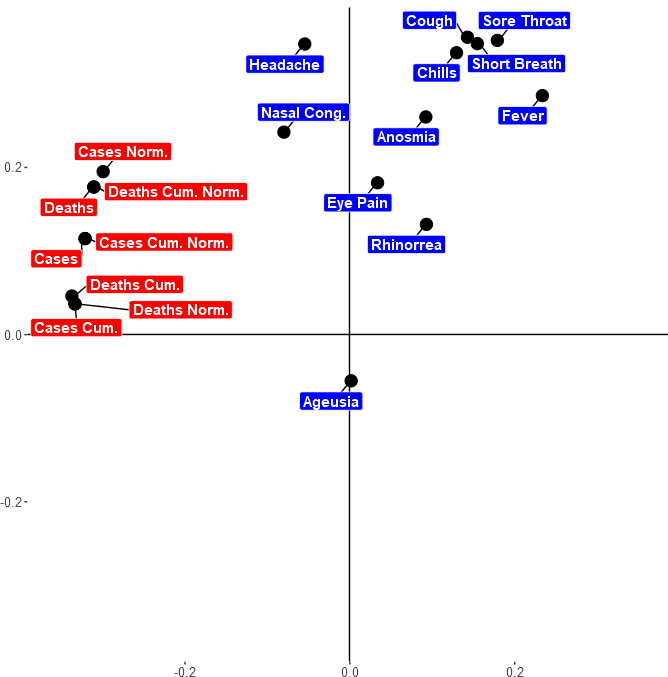


**Scores Plot**

**Component 1**

**Component 2**

**Loadings Plot**

**Component 1**


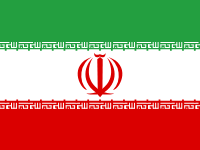


**Component 2**

Figure S44. Principal component analysis scores and loadings plot for Italy. The data starts from 22/Jan/2020 (red dot) to 20/Dec/2020 (Green cross); black dots over red lines indicate weeks.

Figure S45. Principal component analysis scores and loadings plot for South Africa. The data starts from 22/Jan/2020 (red dot) to 20/Dec/2020 (Green cross); black dots over red lines indicate weeks.


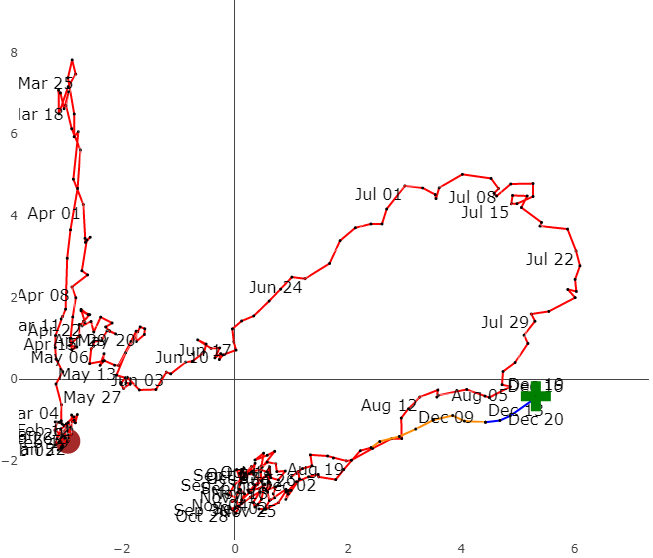

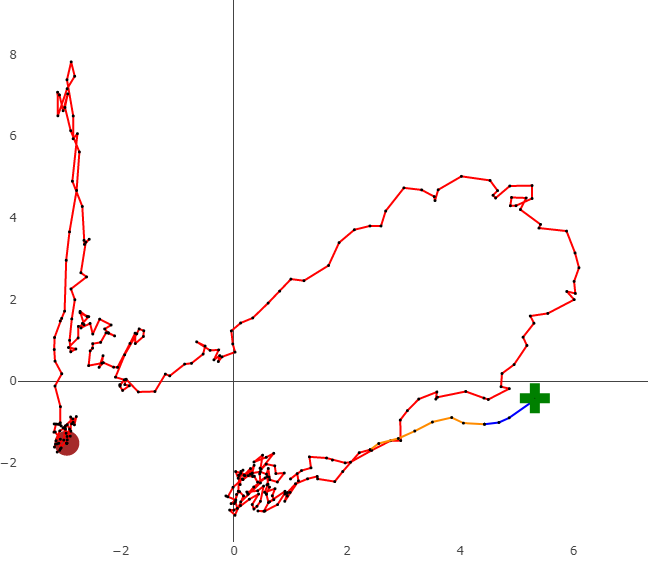

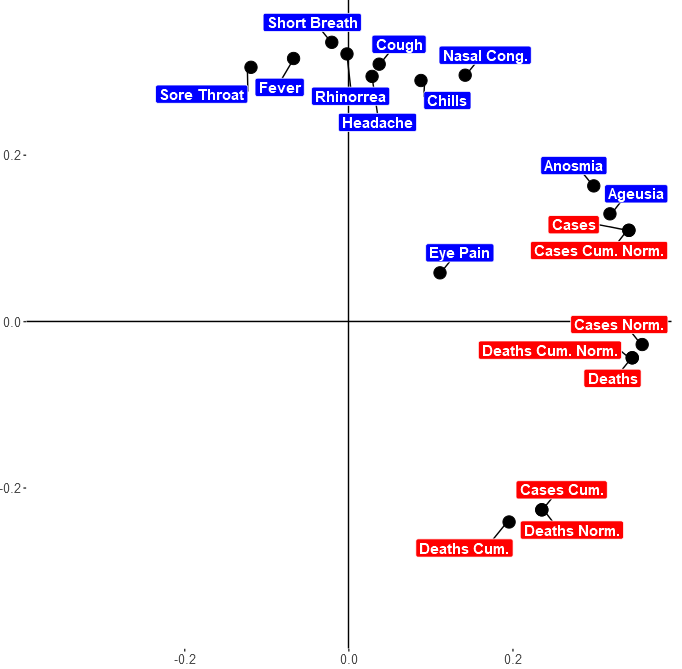


**Scores Plot**

**Component 1**

**Component 2**

**Loadings Plot**

**Component 1**

**Component 2**


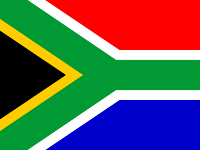


Figure S46. Principal component analysis scores and loadings plot for the United States. The data starts from 22/Jan/2020 (red dot) to 20/Dec/2020 (Green cross); black dots over red lines indicate weeks.

Figure S47. Principal component analysis scores and loadings plot for the United Kingdom. The data starts from 22/Jan/2020 (red dot) to 20/Dec/2020 (Green cross); black dots over red lines indicate weeks.


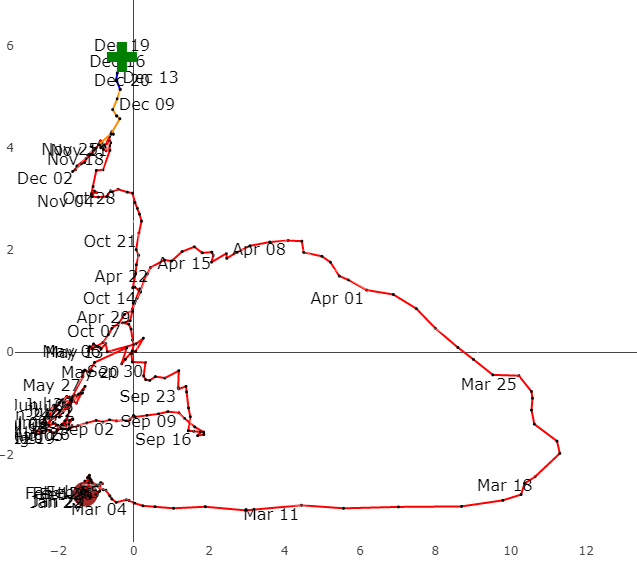

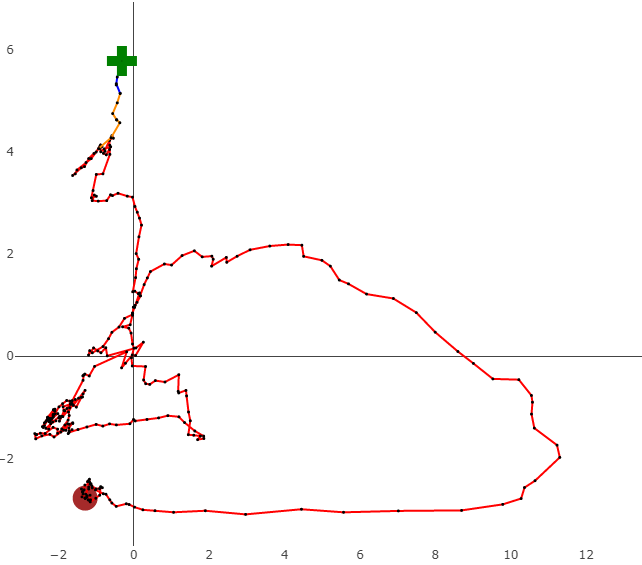

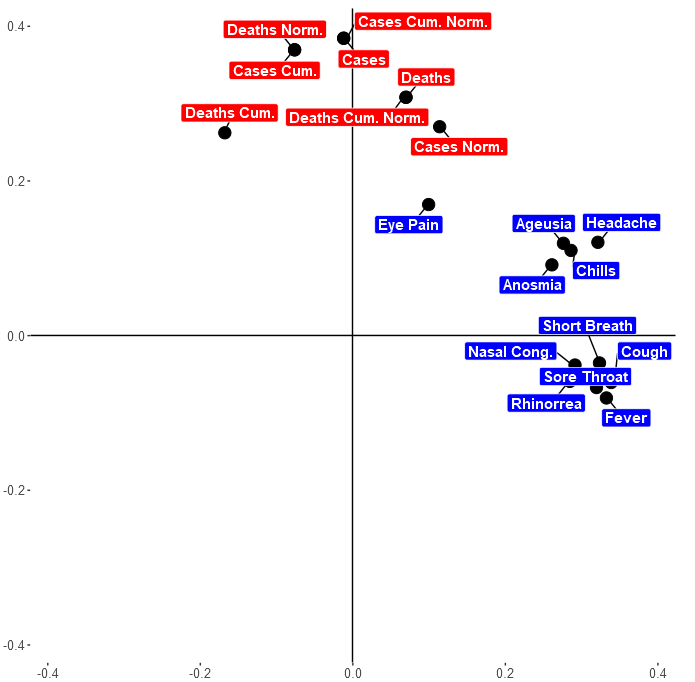


**Scores Plot**

**Component 1**

**Component 2**

**Loadings Plot**

**Component 1**

**Component 2**


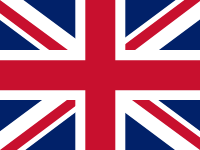


Figure S48. Root-mean-square errors (RMSE) of the prediction error for the principal component 2 of the various models for the selected countries. MA1 and MA7 indicate analyses performed on moving average of data 1 day (i.e., original data) and 7 days, respectively. GT indicates models based on both traditional COVID-19 metrics and Google Trends data, while NOGT models based on COVID-19 metrics only. ARIMA: Autoregressive integrated moving average; ETS: Error Trend Seasonality; NNAR: feed-forward neural network autoregression.

# Supplementary tables

**Table S1. Time-lagged cross-correlations between the 7-day moving average of each topic and the number of new confirmed COVID-19 cases per million population in Australia.**

| **LAG** | **Ageusia** | **Anosmia** | **Chills** | **Cough** | **Eye pain** | **Fever** | **Headache** | **Nasal congestion** | **Rhinorrhea** | **Shortness of breath** | **Sore throat** |
| --- | --- | --- | --- | --- | --- | --- | --- | --- | --- | --- | --- |
| 0 | 0.31 | 0.45 | 0.44 | 0.34 | 0.14 | 0.24 | 0.40 | 0.36 | 0.42 | 0.29 | 0.33 |
| -1 | 0.30 | 0.46 | 0.45 | 0.36 | 0.12 | 0.27 | 0.41 | 0.39 | 0.44 | 0.30 | 0.36 |
| -2 | 0.30 | 0.47 | 0.46 | 0.38 | 0.11 | 0.29 | 0.41 | 0.42 | 0.47 | 0.31 | 0.39 |
| -3 | 0.28 | 0.46 | 0.47 | 0.40 | 0.10 | 0.31 | 0.41 | 0.45 | 0.50 | 0.32 | 0.41 |
| -4 | 0.27 | 0.45 | 0.48 | 0.42 | 0.09 | 0.33 | 0.42 | 0.48 | 0.52 | 0.32 | 0.43 |
| -5 | 0.25 | 0.43 | 0.48 | 0.43 | 0.08 | 0.34 | 0.42 | 0.51 | 0.55 | 0.32 | 0.45 |
| -6 | 0.23 | 0.40 | 0.49 | 0.44 | 0.07 | 0.36 | 0.42 | 0.53 | 0.56 | 0.32 | 0.47 |
| -7 | 0.21 | 0.36 | 0.50 | 0.45 | 0.06 | 0.36 | 0.42 | 0.55 | 0.58 | 0.31 | 0.48 |
| -8 | 0.19 | 0.33 | 0.50 | 0.45 | 0.05 | 0.37 | 0.42 | 0.56 | 0.59 | 0.30 | 0.48 |
| -9 | 0.16 | 0.29 | 0.50 | 0.45 | 0.04 | 0.36 | 0.42 | 0.58 | 0.60 | 0.28 | 0.48 |
| -10 | 0.14 | 0.26 | 0.50 | 0.44 | 0.03 | 0.36 | 0.41 | 0.59 | 0.61 | 0.26 | 0.48 |
| -11 | 0.11 | 0.22 | 0.50 | 0.43 | 0.02 | 0.35 | 0.40 | 0.59 | 0.61 | 0.24 | 0.47 |
| -12 | 0.08 | 0.19 | 0.49 | 0.42 | 0.01 | 0.33 | 0.38 | 0.59 | 0.60 | 0.22 | 0.46 |
| -13 | 0.06 | 0.16 | 0.48 | 0.40 | 0.00 | 0.31 | 0.36 | 0.59 | 0.59 | 0.19 | 0.44 |
| -14 | 0.03 | 0.13 | 0.47 | 0.38 | -0.01 | 0.29 | 0.33 | 0.59 | 0.57 | 0.16 | 0.42 |
| -15 | 0.01 | 0.11 | 0.46 | 0.35 | -0.02 | 0.26 | 0.30 | 0.58 | 0.55 | 0.13 | 0.39 |
| -16 | -0.02 | 0.09 | 0.44 | 0.33 | -0.02 | 0.23 | 0.27 | 0.56 | 0.53 | 0.10 | 0.37 |
| -17 | -0.04 | 0.07 | 0.42 | 0.30 | -0.03 | 0.20 | 0.23 | 0.55 | 0.51 | 0.07 | 0.34 |
| -18 | -0.07 | 0.05 | 0.40 | 0.27 | -0.03 | 0.17 | 0.20 | 0.53 | 0.48 | 0.04 | 0.31 |
| -19 | -0.09 | 0.04 | 0.38 | 0.24 | -0.04 | 0.14 | 0.16 | 0.51 | 0.45 | 0.02 | 0.28 |
| -20 | -0.12 | 0.02 | 0.36 | 0.21 | -0.04 | 0.11 | 0.12 | 0.49 | 0.42 | 0.00 | 0.25 |
| -21 | -0.14 | 0.01 | 0.34 | 0.18 | -0.05 | 0.08 | 0.09 | 0.47 | 0.40 | -0.03 | 0.22 |

Data are presented as Pearson's r. Darker shades indicate higher r values.

**Table S2. Time-lagged cross-correlations between the 7-day moving average of each topic and the number of new confirmed COVID-19 cases per million population in Brazil.**

| **LAG** | **Ageusia** | **Anosmia** | **Chills** | **Cough** | **Eye pain** | **Fever** | **Headache** | **Nasal congestion** | **Rhinorrhea** | **Shortness of breath** | **Sore throat** |
| --- | --- | --- | --- | --- | --- | --- | --- | --- | --- | --- | --- |
| 0 | 0.35 | 0.03 | -0.47 | -0.54 | -0.44 | -0.5 | -0.02 | -0.27 | -0.16 | -0.44 | -0.29 |
| -1 | 0.36 | 0.04 | -0.46 | -0.54 | -0.44 | -0.49 | -0.01 | -0.26 | -0.15 | -0.44 | -0.29 |
| -2 | 0.37 | 0.05 | -0.46 | -0.53 | -0.44 | -0.48 | 0 | -0.26 | -0.15 | -0.43 | -0.29 |
| -3 | 0.38 | 0.07 | -0.45 | -0.53 | -0.44 | -0.48 | 0.02 | -0.25 | -0.14 | -0.42 | -0.29 |
| -4 | 0.39 | 0.08 | -0.45 | -0.53 | -0.43 | -0.47 | 0.03 | -0.25 | -0.14 | -0.42 | -0.28 |
| -5 | 0.4 | 0.09 | -0.45 | -0.53 | -0.43 | -0.47 | 0.04 | -0.24 | -0.14 | -0.41 | -0.28 |
| -6 | 0.41 | 0.1 | -0.44 | -0.53 | -0.43 | -0.46 | 0.06 | -0.24 | -0.14 | -0.4 | -0.28 |
| -7 | 0.42 | 0.11 | -0.45 | -0.52 | -0.43 | -0.45 | 0.07 | -0.24 | -0.14 | -0.39 | -0.27 |
| -8 | 0.43 | 0.12 | -0.45 | -0.52 | -0.43 | -0.45 | 0.08 | -0.23 | -0.14 | -0.39 | -0.27 |
| -9 | 0.44 | 0.13 | -0.45 | -0.52 | -0.42 | -0.44 | 0.1 | -0.23 | -0.14 | -0.38 | -0.27 |
| -10 | 0.44 | 0.14 | -0.45 | -0.51 | -0.42 | -0.43 | 0.11 | -0.23 | -0.14 | -0.37 | -0.27 |
| -11 | 0.45 | 0.15 | -0.45 | -0.51 | -0.42 | -0.42 | 0.12 | -0.23 | -0.14 | -0.36 | -0.27 |
| -12 | 0.46 | 0.16 | -0.45 | -0.51 | -0.41 | -0.42 | 0.13 | -0.23 | -0.14 | -0.35 | -0.27 |
| -13 | 0.47 | 0.16 | -0.44 | -0.51 | -0.41 | -0.41 | 0.14 | -0.22 | -0.14 | -0.35 | -0.27 |
| -14 | 0.48 | 0.17 | -0.43 | -0.5 | -0.4 | -0.4 | 0.15 | -0.22 | -0.14 | -0.34 | -0.27 |
| -15 | 0.48 | 0.18 | -0.41 | -0.5 | -0.39 | -0.39 | 0.15 | -0.21 | -0.13 | -0.33 | -0.27 |
| -16 | 0.49 | 0.19 | -0.4 | -0.5 | -0.38 | -0.38 | 0.16 | -0.21 | -0.11 | -0.32 | -0.27 |
| -17 | 0.49 | 0.2 | -0.38 | -0.5 | -0.36 | -0.38 | 0.16 | -0.21 | -0.1 | -0.31 | -0.27 |
| -18 | 0.5 | 0.21 | -0.37 | -0.49 | -0.35 | -0.37 | 0.17 | -0.21 | -0.09 | -0.3 | -0.27 |
| -19 | 0.5 | 0.22 | -0.35 | -0.49 | -0.34 | -0.37 | 0.17 | -0.21 | -0.08 | -0.29 | -0.27 |
| -20 | 0.5 | 0.23 | -0.33 | -0.49 | -0.32 | -0.36 | 0.18 | -0.21 | -0.08 | -0.28 | -0.27 |
| -21 | 0.5 | 0.24 | -0.32 | -0.48 | -0.31 | -0.36 | 0.18 | -0.21 | -0.07 | -0.28 | -0.27 |

Data are presented as Pearson's r. Darker shades indicate higher r values.

**Table S3. Time-lagged cross-correlations between the 7-day moving average of each topic and the number of new confirmed COVID-19 cases per million population in France.**

| **LAG** | **Ageusia** | **Anosmia** | **Chills** | **Cough** | **Eye pain** | **Fever** | **Headache** | **Nasal congestion** | **Rhinorrhea** | **Shortness of breath** | **Sore throat** |
| --- | --- | --- | --- | --- | --- | --- | --- | --- | --- | --- | --- |
| 0 | 0.53 | 0.23 | -0.19 | -0.1 | -0.15 | -0.14 | 0.28 | 0.21 | -0.05 | -0.16 | 0.01 |
| -1 | 0.53 | 0.24 | -0.2 | -0.1 | -0.15 | -0.14 | 0.28 | 0.22 | -0.04 | -0.16 | 0.03 |
| -2 | 0.54 | 0.24 | -0.2 | -0.09 | -0.15 | -0.13 | 0.27 | 0.24 | -0.03 | -0.15 | 0.04 |
| -3 | 0.55 | 0.25 | -0.2 | -0.09 | -0.15 | -0.12 | 0.27 | 0.26 | -0.01 | -0.15 | 0.06 |
| -4 | 0.55 | 0.25 | -0.21 | -0.08 | -0.15 | -0.12 | 0.27 | 0.27 | 0 | -0.15 | 0.07 |
| -5 | 0.55 | 0.26 | -0.22 | -0.08 | -0.16 | -0.11 | 0.27 | 0.29 | 0.01 | -0.15 | 0.08 |
| -6 | 0.55 | 0.26 | -0.22 | -0.07 | -0.17 | -0.1 | 0.26 | 0.31 | 0.03 | -0.15 | 0.1 |
| -7 | 0.55 | 0.26 | -0.22 | -0.07 | -0.17 | -0.1 | 0.26 | 0.33 | 0.04 | -0.15 | 0.11 |
| -8 | 0.54 | 0.26 | -0.22 | -0.06 | -0.16 | -0.09 | 0.25 | 0.34 | 0.05 | -0.15 | 0.12 |
| -9 | 0.54 | 0.25 | -0.23 | -0.06 | -0.16 | -0.09 | 0.25 | 0.35 | 0.07 | -0.15 | 0.13 |
| -10 | 0.53 | 0.25 | -0.23 | -0.05 | -0.17 | -0.08 | 0.24 | 0.37 | 0.08 | -0.14 | 0.15 |
| -11 | 0.51 | 0.24 | -0.23 | -0.05 | -0.18 | -0.08 | 0.23 | 0.38 | 0.09 | -0.14 | 0.16 |
| -12 | 0.5 | 0.23 | -0.23 | -0.04 | -0.18 | -0.07 | 0.23 | 0.39 | 0.1 | -0.13 | 0.17 |
| -13 | 0.49 | 0.23 | -0.23 | -0.04 | -0.18 | -0.07 | 0.22 | 0.39 | 0.1 | -0.13 | 0.18 |
| -14 | 0.49 | 0.23 | -0.23 | -0.04 | -0.18 | -0.06 | 0.21 | 0.4 | 0.11 | -0.13 | 0.18 |
| -15 | 0.48 | 0.23 | -0.23 | -0.04 | -0.2 | -0.06 | 0.2 | 0.4 | 0.12 | -0.13 | 0.19 |
| -16 | 0.48 | 0.24 | -0.24 | -0.04 | -0.21 | -0.06 | 0.19 | 0.41 | 0.12 | -0.13 | 0.2 |
| -17 | 0.48 | 0.25 | -0.25 | -0.04 | -0.21 | -0.06 | 0.19 | 0.41 | 0.13 | -0.13 | 0.21 |
| -18 | 0.48 | 0.26 | -0.26 | -0.04 | -0.21 | -0.06 | 0.18 | 0.42 | 0.13 | -0.14 | 0.22 |
| -19 | 0.48 | 0.27 | -0.26 | -0.04 | -0.22 | -0.06 | 0.17 | 0.42 | 0.14 | -0.14 | 0.22 |
| -20 | 0.47 | 0.28 | -0.27 | -0.04 | -0.23 | -0.06 | 0.16 | 0.42 | 0.15 | -0.15 | 0.23 |
| -21 | 0.46 | 0.29 | -0.27 | -0.04 | -0.23 | -0.05 | 0.15 | 0.43 | 0.16 | -0.15 | 0.24 |

Data are presented as Pearson's r. Darker shades indicate higher r values.

**Table S4. Time-lagged cross-correlations between the 7-day moving average of each topic and the number of new confirmed COVID-19 cases per million population in India.**

| **LAG** | **Ageusia** | **Anosmia** | **Chills** | **Cough** | **Eye pain** | **Fever** | **Headache** | **Nasal congestion** | **Rhinorrhea** | **Shortness of breath** | **Sore throat** |
| --- | --- | --- | --- | --- | --- | --- | --- | --- | --- | --- | --- |
| 0 | 0.89 | 0.91 | 0.19 | -0.37 | 0.47 | 0.52 | 0.23 | -0.48 | -0.12 | -0.22 | -0.29 |
| -1 | 0.9 | 0.92 | 0.2 | -0.38 | 0.48 | 0.53 | 0.25 | -0.47 | -0.12 | -0.21 | -0.29 |
| -2 | 0.9 | 0.92 | 0.2 | -0.38 | 0.49 | 0.54 | 0.26 | -0.47 | -0.12 | -0.2 | -0.28 |
| -3 | 0.9 | 0.92 | 0.21 | -0.38 | 0.49 | 0.55 | 0.27 | -0.47 | -0.13 | -0.19 | -0.28 |
| -4 | 0.9 | 0.93 | 0.21 | -0.38 | 0.5 | 0.55 | 0.28 | -0.47 | -0.13 | -0.18 | -0.27 |
| -5 | 0.91 | 0.93 | 0.22 | -0.39 | 0.5 | 0.56 | 0.3 | -0.47 | -0.13 | -0.18 | -0.27 |
| -6 | 0.91 | 0.93 | 0.22 | -0.39 | 0.51 | 0.57 | 0.31 | -0.47 | -0.13 | -0.17 | -0.26 |
| -7 | 0.91 | 0.93 | 0.22 | -0.39 | 0.52 | 0.57 | 0.32 | -0.47 | -0.13 | -0.16 | -0.26 |
| -8 | 0.91 | 0.93 | 0.23 | -0.39 | 0.52 | 0.57 | 0.33 | -0.47 | -0.13 | -0.15 | -0.25 |
| -9 | 0.91 | 0.93 | 0.23 | -0.4 | 0.53 | 0.58 | 0.34 | -0.47 | -0.14 | -0.14 | -0.25 |
| -10 | 0.91 | 0.93 | 0.23 | -0.4 | 0.54 | 0.58 | 0.34 | -0.47 | -0.14 | -0.13 | -0.24 |
| -11 | 0.91 | 0.92 | 0.24 | -0.41 | 0.55 | 0.58 | 0.35 | -0.47 | -0.14 | -0.12 | -0.24 |
| -12 | 0.9 | 0.92 | 0.24 | -0.41 | 0.55 | 0.59 | 0.36 | -0.47 | -0.14 | -0.11 | -0.23 |
| -13 | 0.9 | 0.92 | 0.24 | -0.41 | 0.56 | 0.59 | 0.37 | -0.47 | -0.14 | -0.11 | -0.23 |
| -14 | 0.9 | 0.91 | 0.25 | -0.42 | 0.57 | 0.59 | 0.38 | -0.47 | -0.14 | -0.1 | -0.23 |
| -15 | 0.9 | 0.91 | 0.25 | -0.42 | 0.57 | 0.59 | 0.38 | -0.47 | -0.14 | -0.09 | -0.22 |
| -16 | 0.89 | 0.9 | 0.25 | -0.43 | 0.58 | 0.59 | 0.39 | -0.47 | -0.15 | -0.08 | -0.22 |
| -17 | 0.89 | 0.9 | 0.25 | -0.43 | 0.58 | 0.59 | 0.39 | -0.46 | -0.15 | -0.07 | -0.21 |
| -18 | 0.89 | 0.89 | 0.26 | -0.44 | 0.58 | 0.59 | 0.4 | -0.46 | -0.15 | -0.06 | -0.21 |
| -19 | 0.88 | 0.89 | 0.26 | -0.44 | 0.59 | 0.59 | 0.4 | -0.46 | -0.15 | -0.05 | -0.21 |
| -20 | 0.88 | 0.88 | 0.26 | -0.45 | 0.59 | 0.59 | 0.41 | -0.46 | -0.16 | -0.04 | -0.2 |
| -21 | 0.88 | 0.88 | 0.27 | -0.45 | 0.59 | 0.59 | 0.41 | -0.46 | -0.16 | -0.04 | -0.2 |

Data are presented as Pearson's r. Darker shades indicate higher r values.

**Table S5. Time-lagged cross-correlations between the 7-day moving average of each topic and the number of new confirmed COVID-19 cases per million population in Iran.**

| **LAG** | **Ageusia** | **Anosmia** | **Chills** | **Cough** | **Eye pain** | **Fever** | **Headache** | **Nasal congestion** | **Rhinorrhea** | **Shortness of breath** | **Sore throat** |
| --- | --- | --- | --- | --- | --- | --- | --- | --- | --- | --- | --- |
| 0 | -0.14 | -0.18 | -0.1 | -0.06 | -0.2 | -0.42 | 0.3 | 0.23 | -0.11 | -0.16 | -0.19 |
| -1 | -0.14 | -0.17 | -0.09 | -0.04 | -0.2 | -0.41 | 0.32 | 0.25 | -0.11 | -0.15 | -0.17 |
| -2 | -0.14 | -0.16 | -0.07 | -0.03 | -0.19 | -0.4 | 0.33 | 0.27 | -0.11 | -0.14 | -0.16 |
| -3 | -0.14 | -0.14 | -0.05 | -0.01 | -0.19 | -0.39 | 0.34 | 0.29 | -0.11 | -0.13 | -0.14 |
| -4 | -0.14 | -0.13 | -0.04 | 0 | -0.18 | -0.38 | 0.36 | 0.31 | -0.11 | -0.13 | -0.13 |
| -5 | -0.13 | -0.12 | -0.03 | 0.02 | -0.18 | -0.37 | 0.37 | 0.33 | -0.1 | -0.12 | -0.11 |
| -6 | -0.13 | -0.11 | -0.01 | 0.03 | -0.17 | -0.36 | 0.38 | 0.35 | -0.1 | -0.12 | -0.1 |
| -7 | -0.13 | -0.1 | 0 | 0.04 | -0.17 | -0.35 | 0.39 | 0.36 | -0.09 | -0.11 | -0.08 |
| -8 | -0.13 | -0.09 | 0.02 | 0.06 | -0.16 | -0.34 | 0.4 | 0.38 | -0.08 | -0.1 | -0.07 |
| -9 | -0.13 | -0.08 | 0.03 | 0.07 | -0.15 | -0.33 | 0.41 | 0.4 | -0.08 | -0.1 | -0.06 |
| -10 | -0.13 | -0.07 | 0.05 | 0.08 | -0.14 | -0.32 | 0.41 | 0.41 | -0.07 | -0.09 | -0.04 |
| -11 | -0.13 | -0.06 | 0.06 | 0.09 | -0.13 | -0.31 | 0.42 | 0.43 | -0.06 | -0.09 | -0.03 |
| -12 | -0.13 | -0.05 | 0.07 | 0.1 | -0.12 | -0.31 | 0.42 | 0.44 | -0.06 | -0.09 | -0.02 |
| -13 | -0.13 | -0.04 | 0.09 | 0.11 | -0.11 | -0.3 | 0.43 | 0.46 | -0.06 | -0.08 | -0.01 |
| -14 | -0.13 | -0.03 | 0.1 | 0.11 | -0.11 | -0.29 | 0.43 | 0.47 | -0.05 | -0.08 | 0 |
| -15 | -0.13 | -0.02 | 0.11 | 0.12 | -0.1 | -0.29 | 0.43 | 0.48 | -0.05 | -0.08 | 0.01 |
| -16 | -0.13 | 0 | 0.12 | 0.13 | -0.1 | -0.28 | 0.44 | 0.5 | -0.04 | -0.08 | 0.01 |
| -17 | -0.12 | 0.01 | 0.14 | 0.13 | -0.09 | -0.27 | 0.44 | 0.51 | -0.04 | -0.08 | 0.02 |
| -18 | -0.12 | 0.02 | 0.15 | 0.13 | -0.09 | -0.27 | 0.43 | 0.52 | -0.03 | -0.08 | 0.02 |
| -19 | -0.12 | 0.03 | 0.16 | 0.13 | -0.09 | -0.26 | 0.43 | 0.53 | -0.02 | -0.08 | 0.03 |
| -20 | -0.12 | 0.04 | 0.16 | 0.13 | -0.09 | -0.25 | 0.43 | 0.54 | -0.02 | -0.09 | 0.03 |
| -21 | -0.12 | 0.05 | 0.17 | 0.13 | -0.09 | -0.25 | 0.43 | 0.55 | -0.01 | -0.09 | 0.03 |

Data are presented as Pearson's r. Darker shades indicate higher r values.

**Table S6. Time-lagged cross-correlations between the 7-day moving average of each topic and the number of new confirmed COVID-19 cases per million population in Italy.**

| **LAG** | **Ageusia** | **Anosmia** | **Chills** | **Cough** | **Eye pain** | **Fever** | **Headache** | **Nasal congestion** | **Rhinorrhea** | **Shortness of breath** | **Sore throat** |
| --- | --- | --- | --- | --- | --- | --- | --- | --- | --- | --- | --- |
| 0 | 0.69 | 0.49 | 0.09 | 0.08 | 0.07 | 0.17 | 0.81 | 0.24 | 0 | -0.02 | 0.22 |
| -1 | 0.71 | 0.51 | 0.09 | 0.09 | 0.08 | 0.19 | 0.82 | 0.24 | 0.01 | -0.01 | 0.23 |
| -2 | 0.72 | 0.53 | 0.1 | 0.1 | 0.08 | 0.21 | 0.84 | 0.25 | 0.01 | 0 | 0.24 |
| -3 | 0.74 | 0.54 | 0.1 | 0.12 | 0.09 | 0.23 | 0.85 | 0.25 | 0.01 | 0 | 0.25 |
| -4 | 0.76 | 0.56 | 0.1 | 0.13 | 0.1 | 0.24 | 0.86 | 0.25 | 0.02 | 0.01 | 0.26 |
| -5 | 0.77 | 0.57 | 0.11 | 0.14 | 0.1 | 0.26 | 0.87 | 0.25 | 0.02 | 0.01 | 0.27 |
| -6 | 0.78 | 0.59 | 0.11 | 0.15 | 0.11 | 0.27 | 0.87 | 0.25 | 0.02 | 0.02 | 0.28 |
| -7 | 0.79 | 0.6 | 0.11 | 0.16 | 0.11 | 0.29 | 0.87 | 0.26 | 0.02 | 0.02 | 0.29 |
| -8 | 0.8 | 0.61 | 0.11 | 0.17 | 0.11 | 0.3 | 0.87 | 0.26 | 0.02 | 0.03 | 0.3 |
| -9 | 0.8 | 0.61 | 0.11 | 0.17 | 0.11 | 0.31 | 0.87 | 0.26 | 0.02 | 0.03 | 0.31 |
| -10 | 0.81 | 0.62 | 0.11 | 0.18 | 0.12 | 0.32 | 0.86 | 0.26 | 0.02 | 0.03 | 0.32 |
| -11 | 0.81 | 0.62 | 0.11 | 0.19 | 0.12 | 0.33 | 0.86 | 0.26 | 0.02 | 0.03 | 0.33 |
| -12 | 0.81 | 0.62 | 0.11 | 0.19 | 0.12 | 0.34 | 0.85 | 0.26 | 0.02 | 0.03 | 0.34 |
| -13 | 0.81 | 0.62 | 0.11 | 0.2 | 0.13 | 0.34 | 0.85 | 0.26 | 0.02 | 0.03 | 0.36 |
| -14 | 0.8 | 0.62 | 0.11 | 0.2 | 0.13 | 0.35 | 0.84 | 0.25 | 0.02 | 0.03 | 0.37 |
| -15 | 0.8 | 0.62 | 0.1 | 0.21 | 0.13 | 0.35 | 0.83 | 0.25 | 0.02 | 0.03 | 0.38 |
| -16 | 0.79 | 0.62 | 0.1 | 0.21 | 0.13 | 0.36 | 0.82 | 0.24 | 0.02 | 0.03 | 0.4 |
| -17 | 0.78 | 0.61 | 0.09 | 0.21 | 0.13 | 0.36 | 0.8 | 0.23 | 0.02 | 0.02 | 0.41 |
| -18 | 0.77 | 0.6 | 0.09 | 0.21 | 0.12 | 0.36 | 0.79 | 0.23 | 0.02 | 0.02 | 0.42 |
| -19 | 0.75 | 0.6 | 0.08 | 0.21 | 0.12 | 0.36 | 0.77 | 0.22 | 0.02 | 0.01 | 0.44 |
| -20 | 0.74 | 0.58 | 0.08 | 0.21 | 0.11 | 0.36 | 0.75 | 0.22 | 0.02 | 0.01 | 0.45 |
| -21 | 0.72 | 0.57 | 0.07 | 0.21 | 0.11 | 0.36 | 0.73 | 0.21 | 0.02 | 0 | 0.46 |

Data are presented as Pearson's r. Darker shades indicate higher r values.

**Table S7. Time-lagged cross-correlations between the 7-day moving average of each topic and the number of new confirmed COVID-19 cases per million population in South Africa.**

| **LAG** | **Ageusia** | **Anosmia** | **Chills** | **Cough** | **Eye pain** | **Fever** | **Headache** | **Nasal congestion** | **Rhinorrhea** | **Shortness of breath** | **Sore throat** |
| --- | --- | --- | --- | --- | --- | --- | --- | --- | --- | --- | --- |
| 0 | 0.91 | 0.92 | 0.45 | 0.16 | 0.26 | 0.02 | 0.34 | 0.45 | 0.3 | 0.19 | -0.15 |
| -1 | 0.91 | 0.91 | 0.46 | 0.17 | 0.27 | 0.02 | 0.35 | 0.46 | 0.3 | 0.19 | -0.14 |
| -2 | 0.9 | 0.91 | 0.48 | 0.17 | 0.29 | 0.02 | 0.35 | 0.46 | 0.3 | 0.19 | -0.13 |
| -3 | 0.9 | 0.91 | 0.49 | 0.17 | 0.3 | 0.03 | 0.34 | 0.47 | 0.29 | 0.19 | -0.13 |
| -4 | 0.89 | 0.91 | 0.5 | 0.17 | 0.31 | 0.03 | 0.34 | 0.48 | 0.29 | 0.18 | -0.12 |
| -5 | 0.88 | 0.9 | 0.51 | 0.17 | 0.32 | 0.03 | 0.33 | 0.48 | 0.28 | 0.18 | -0.11 |
| -6 | 0.88 | 0.9 | 0.52 | 0.17 | 0.33 | 0.03 | 0.32 | 0.49 | 0.28 | 0.18 | -0.11 |
| -7 | 0.87 | 0.89 | 0.53 | 0.17 | 0.34 | 0.03 | 0.31 | 0.49 | 0.27 | 0.17 | -0.11 |
| -8 | 0.86 | 0.88 | 0.54 | 0.16 | 0.35 | 0.03 | 0.3 | 0.49 | 0.27 | 0.16 | -0.1 |
| -9 | 0.85 | 0.87 | 0.55 | 0.16 | 0.35 | 0.03 | 0.29 | 0.49 | 0.26 | 0.16 | -0.1 |
| -10 | 0.84 | 0.86 | 0.55 | 0.16 | 0.35 | 0.03 | 0.29 | 0.48 | 0.25 | 0.14 | -0.1 |
| -11 | 0.83 | 0.84 | 0.56 | 0.15 | 0.35 | 0.03 | 0.28 | 0.48 | 0.23 | 0.13 | -0.1 |
| -12 | 0.81 | 0.83 | 0.56 | 0.15 | 0.34 | 0.03 | 0.27 | 0.47 | 0.22 | 0.13 | -0.1 |
| -13 | 0.8 | 0.81 | 0.56 | 0.14 | 0.34 | 0.03 | 0.26 | 0.46 | 0.21 | 0.12 | -0.1 |
| -14 | 0.78 | 0.79 | 0.56 | 0.13 | 0.33 | 0.03 | 0.26 | 0.45 | 0.19 | 0.11 | -0.11 |
| -15 | 0.76 | 0.77 | 0.55 | 0.12 | 0.32 | 0.02 | 0.25 | 0.44 | 0.18 | 0.1 | -0.11 |
| -16 | 0.74 | 0.75 | 0.55 | 0.11 | 0.31 | 0.02 | 0.25 | 0.43 | 0.16 | 0.09 | -0.11 |
| -17 | 0.72 | 0.73 | 0.55 | 0.1 | 0.29 | 0.02 | 0.25 | 0.41 | 0.15 | 0.08 | -0.11 |
| -18 | 0.7 | 0.7 | 0.54 | 0.09 | 0.27 | 0.02 | 0.24 | 0.4 | 0.14 | 0.08 | -0.12 |
| -19 | 0.67 | 0.68 | 0.54 | 0.08 | 0.25 | 0.02 | 0.24 | 0.39 | 0.12 | 0.07 | -0.12 |
| -20 | 0.65 | 0.66 | 0.53 | 0.07 | 0.24 | 0.01 | 0.24 | 0.38 | 0.11 | 0.05 | -0.12 |
| -21 | 0.62 | 0.64 | 0.52 | 0.06 | 0.23 | 0.01 | 0.23 | 0.36 | 0.1 | 0.04 | -0.13 |

Data are presented as Pearson's r. Darker shades indicate higher r values.

**Table S8. Time-lagged cross-correlations between the 7-day moving average of each topic and the number of new confirmed COVID-19 cases per million population in the United Kingdom.**

| **LAG** | **Ageusia** | **Anosmia** | **Chills** | **Cough** | **Eye pain** | **Fever** | **Headache** | **Nasal congestion** | **Rhinorrhea** | **Shortness of breath** | **Sore throat** |
| --- | --- | --- | --- | --- | --- | --- | --- | --- | --- | --- | --- |
| 0 | 0.17 | 0.07 | 0.15 | -0.13 | 0.32 | -0.13 | 0.07 | -0.07 | -0.09 | -0.09 | -0.09 |
| -1 | 0.18 | 0.07 | 0.15 | -0.12 | 0.33 | -0.13 | 0.07 | -0.06 | -0.08 | -0.08 | -0.08 |
| -2 | 0.18 | 0.07 | 0.15 | -0.11 | 0.33 | -0.12 | 0.07 | -0.05 | -0.07 | -0.08 | -0.07 |
| -3 | 0.18 | 0.08 | 0.16 | -0.11 | 0.33 | -0.11 | 0.08 | -0.04 | -0.06 | -0.07 | -0.06 |
| -4 | 0.19 | 0.08 | 0.17 | -0.1 | 0.32 | -0.11 | 0.08 | -0.02 | -0.05 | -0.07 | -0.05 |
| -5 | 0.19 | 0.08 | 0.17 | -0.09 | 0.3 | -0.1 | 0.08 | -0.01 | -0.04 | -0.06 | -0.03 |
| -6 | 0.2 | 0.09 | 0.18 | -0.08 | 0.3 | -0.09 | 0.08 | 0.01 | -0.02 | -0.06 | -0.02 |
| -7 | 0.2 | 0.09 | 0.18 | -0.07 | 0.3 | -0.08 | 0.08 | 0.02 | -0.01 | -0.05 | -0.01 |
| -8 | 0.2 | 0.09 | 0.18 | -0.06 | 0.29 | -0.07 | 0.08 | 0.04 | 0 | -0.05 | 0 |
| -9 | 0.21 | 0.1 | 0.18 | -0.06 | 0.28 | -0.07 | 0.08 | 0.05 | 0.01 | -0.05 | 0.01 |
| -10 | 0.21 | 0.1 | 0.17 | -0.05 | 0.27 | -0.06 | 0.08 | 0.07 | 0.03 | -0.05 | 0.03 |
| -11 | 0.2 | 0.1 | 0.17 | -0.04 | 0.26 | -0.05 | 0.08 | 0.08 | 0.04 | -0.04 | 0.04 |
| -12 | 0.2 | 0.1 | 0.17 | -0.03 | 0.25 | -0.04 | 0.08 | 0.09 | 0.05 | -0.04 | 0.05 |
| -13 | 0.2 | 0.09 | 0.18 | -0.02 | 0.24 | -0.04 | 0.08 | 0.1 | 0.07 | -0.04 | 0.06 |
| -14 | 0.2 | 0.09 | 0.19 | -0.01 | 0.24 | -0.03 | 0.08 | 0.11 | 0.08 | -0.04 | 0.07 |
| -15 | 0.2 | 0.09 | 0.2 | -0.01 | 0.24 | -0.02 | 0.08 | 0.12 | 0.1 | -0.04 | 0.09 |
| -16 | 0.2 | 0.09 | 0.21 | 0 | 0.24 | -0.02 | 0.08 | 0.14 | 0.11 | -0.04 | 0.1 |
| -17 | 0.19 | 0.09 | 0.22 | 0.01 | 0.24 | -0.01 | 0.08 | 0.15 | 0.13 | -0.04 | 0.11 |
| -18 | 0.19 | 0.09 | 0.23 | 0.01 | 0.24 | 0 | 0.08 | 0.16 | 0.14 | -0.04 | 0.12 |
| -19 | 0.19 | 0.08 | 0.23 | 0.02 | 0.24 | 0 | 0.08 | 0.17 | 0.15 | -0.04 | 0.13 |
| -20 | 0.19 | 0.08 | 0.23 | 0.02 | 0.23 | 0.01 | 0.08 | 0.18 | 0.17 | -0.04 | 0.14 |
| -21 | 0.18 | 0.08 | 0.22 | 0.03 | 0.21 | 0.01 | 0.07 | 0.19 | 0.18 | -0.04 | 0.15 |

Data are presented as Pearson's r. Darker shades indicate higher r values.

**Table S9. Time-lagged cross-correlations between the 7-day moving average of each topic and the number of new confirmed COVID-19 cases per million population in the United States.**

| **LAG** | **Ageusia** | **Anosmia** | **Chills** | **Cough** | **Eye pain** | **Fever** | **Headache** | **Nasal congestion** | **Rhinorrhea** | **Shortness of breath** | **Sore throat** |
| --- | --- | --- | --- | --- | --- | --- | --- | --- | --- | --- | --- |
| 0 | 0.84 | 0.43 | -0.26 | -0.32 | 0.57 | -0.27 | 0.25 | 0.21 | -0.12 | -0.29 | -0.22 |
| -1 | 0.83 | 0.43 | -0.26 | -0.32 | 0.58 | -0.27 | 0.25 | 0.21 | -0.11 | -0.28 | -0.21 |
| -2 | 0.82 | 0.43 | -0.26 | -0.31 | 0.59 | -0.27 | 0.25 | 0.21 | -0.11 | -0.28 | -0.21 |
| -3 | 0.81 | 0.44 | -0.26 | -0.31 | 0.59 | -0.26 | 0.24 | 0.21 | -0.1 | -0.28 | -0.2 |
| -4 | 0.81 | 0.44 | -0.27 | -0.31 | 0.59 | -0.26 | 0.24 | 0.21 | -0.1 | -0.27 | -0.2 |
| -5 | 0.8 | 0.44 | -0.27 | -0.3 | 0.58 | -0.25 | 0.23 | 0.21 | -0.09 | -0.27 | -0.19 |
| -6 | 0.79 | 0.43 | -0.27 | -0.3 | 0.57 | -0.25 | 0.23 | 0.2 | -0.09 | -0.27 | -0.18 |
| -7 | 0.79 | 0.43 | -0.27 | -0.3 | 0.55 | -0.25 | 0.22 | 0.2 | -0.08 | -0.26 | -0.18 |
| -8 | 0.78 | 0.42 | -0.27 | -0.3 | 0.52 | -0.24 | 0.21 | 0.2 | -0.08 | -0.26 | -0.17 |
| -9 | 0.77 | 0.42 | -0.28 | -0.3 | 0.51 | -0.24 | 0.2 | 0.2 | -0.08 | -0.26 | -0.17 |
| -10 | 0.76 | 0.41 | -0.28 | -0.29 | 0.49 | -0.24 | 0.19 | 0.19 | -0.07 | -0.26 | -0.17 |
| -11 | 0.75 | 0.4 | -0.29 | -0.29 | 0.47 | -0.23 | 0.19 | 0.19 | -0.07 | -0.26 | -0.16 |
| -12 | 0.74 | 0.39 | -0.29 | -0.29 | 0.45 | -0.23 | 0.17 | 0.18 | -0.06 | -0.25 | -0.16 |
| -13 | 0.73 | 0.38 | -0.29 | -0.29 | 0.43 | -0.23 | 0.17 | 0.18 | -0.06 | -0.25 | -0.16 |
| -14 | 0.71 | 0.37 | -0.3 | -0.28 | 0.41 | -0.23 | 0.16 | 0.17 | -0.06 | -0.25 | -0.15 |
| -15 | 0.7 | 0.36 | -0.3 | -0.28 | 0.4 | -0.23 | 0.15 | 0.17 | -0.06 | -0.25 | -0.15 |
| -16 | 0.68 | 0.35 | -0.3 | -0.28 | 0.38 | -0.23 | 0.14 | 0.16 | -0.06 | -0.25 | -0.15 |
| -17 | 0.66 | 0.33 | -0.31 | -0.28 | 0.37 | -0.23 | 0.13 | 0.15 | -0.06 | -0.25 | -0.15 |
| -18 | 0.64 | 0.32 | -0.31 | -0.28 | 0.36 | -0.23 | 0.12 | 0.15 | -0.06 | -0.25 | -0.15 |
| -19 | 0.62 | 0.31 | -0.31 | -0.28 | 0.37 | -0.23 | 0.11 | 0.14 | -0.06 | -0.25 | -0.15 |
| -20 | 0.6 | 0.3 | -0.31 | -0.28 | 0.37 | -0.23 | 0.11 | 0.13 | -0.06 | -0.25 | -0.15 |
| -21 | 0.58 | 0.29 | -0.32 | -0.28 | 0.38 | -0.23 | 0.1 | 0.12 | -0.07 | -0.25 | -0.15 |

Data are presented as Pearson's r. Darker shades indicate higher r values.
